# Demand and supply analysis for maternal and child health services at the primary healthcare level in Nigeria

**DOI:** 10.1186/s12913-023-10210-6

**Published:** 2023-11-21

**Authors:** Udochukwu U. Ogu, Bassey Ebenso, Tolib Mirzoev, Nkolika Uguru, Enyi Etiaba, Benjamin Uzochukwu, Nkoli Ezumah, Obinna Onwujekwe

**Affiliations:** 1 Department of Pharmacology and Therapeutics, College of Medicine, Health Policy Research Group, University of Nigeria, Enugu, 400001 Nigeria; 2 University of Leeds, Leeds, UK; 3 London School of Hygiene and Tropical Medicine, London, UK; 4 Department of Health Administration and Management, College of Medicine, University of Nigeria, Enugu, 400001 Nigeria; 5 Faculty of Dentistry, College of Medicine, University of Nigeria, Enugu, 400001 Nigeria; 6 Department of Community Medicine, College of Medicine, University of Nigeria, Enugu, 400001 Nigeria

**Keywords:** Maternal and child health, Demand and supply, Three-delays model, Primary healthcare

## Abstract

**Background:**

The low demand for maternal and child health services is a significant factor in Nigeria's high maternal death rate. This paper explores demand and supply-side determinants at the primary healthcare level, highlighting factors affecting provision and utilization.

**Methods:**

This qualitative study was undertaken in Anambra state, southeast Nigeria. Anambra state was purposively chosen because a maternal and child health programme had just been implemented in the state. The three-delay model was used to analyze supply and demand factors that affect MCH services and improve access to care for pregnant women/mothers and newborns/infants.

**Result:**

The findings show that there were problems with both the demand and supply aspects of the programme and both were interlinked. For service users, their delays were connected to the constraints on the supply side. On the demand side, the delays include poor conditions of the facilities, the roads to the facilities are inaccessible, and equipment were lacking in the facilities. These delayed the utilisation of facilities. On the supply side, the delays include the absence of security (fence, security guard), poor citing of the facilities, inadequate accommodation, no emergency transport for referrals, and lack of trained staff to man equipment. These delayed the provision of services.

**Conclusion:**

Our findings show that there were problems with both the demand and supply aspects of the programme, and both were interlinked. For service users, their delays were connected to the constraints on the supply side.

## Introduction

Globally, more than 500,000 women lose their lives in the process of reproduction [1] and in in 2018 an estimated 15,000 children died before the age of five daily, accounting for 47% of all under-five deaths in 2018 [2]. However, United Nation (UN) inter-agency showed that the global maternal mortality ratio (MMR) declined by 34% between 2000 and 2020, from 342 to 223 deaths per 100,000 live births, resulting in an average drop of 2.1% annually in MMR per year [3]. Nigeria still has one of the highest maternal mortality ratios in the world (512 deaths per 100,000 live births) and infant mortality ratios (56 per 1,000 live births) irrespective of efforts that have been made [1, 4, 5]. About 40,000 maternal deaths occur in Nigeria every year i.e. about 14% of the global percentage [6]. The low level of utilization (demand) of maternal and child health (MCH) services is a major factor that is responsible for the high maternal mortality in most parts of Nigeria [1].

Proper provision and timely utilization of MCH services is one major way to improve health outcomes and reduce maternal and infant mortality. Although the utilization of appropriate maternal health services are associated with improvements in maternal and neonatal health outcomes [7–9], only about 36% of births take place in formal health facilities in Nigeria while about 63% of women give birth at home [10, 11]. The low numbers of women who give birth in formal health facilities have been linked to constraints with the supply and demand of MCH services in many low and middle-income countries (LMICs) such as Nigeria.

Understanding the demand and supply issues that constrain the optimal supply and utilization of MCH services will programmatically help in improving access and use of such services, with positive effects on MCH outcomes. Prior studies [12–15] have offered insights into factors that influence (either positively or negatively) the demand (utilization) and supply (provision) of MCH services. A study found that education increased, significantly, the use of a hospital or a maternity home for prenatal care, delivery, and post-natal care [12]. Another study found that the presence of a male security guard in the facility, staff accommodation, perimeter fencing, and lighting areimportant factors that facilitated the provision of services [15].

Generally, factors that have been found to influence the utilization of MCH services are education, maternal age, perception of need, care preference,, distance, cultural beliefs, and cost of services [15–19]. Also, factors that have been found to influence the provision of MCH services are the structural nature of the health facility, presence/lack of equipment, supplies, drugs, inadequate staff, staff accommodation, and adequate payment of salaries [9, 14, 15, 19]. There is also a link between utilization and provision of MCH services—improved maternal and neonatal outcomes, since health education is an important component of these services [1, 16].

To improve the supply and demand for MCH services, the Federal government of Nigeria introduced the Subsidy Reinvestment Programme (SURE-P) maternal and child programme (SURE-P/MCH) between 2012 and 2015 [20]. The programme was designed to ensure access to maternal services and improvement in the quality of maternal health care by (for the demand component) using a conditional cash transfer (CCT) scheme as a resource. CCTs were given to those who registered at designated primary healthcare (PHC) facilities, received four ANC check-ups, gave birth at participating health facilities and had their infants receive the first series of vaccinations at these facilities [21]. The supply component aimed to enhance access to quality health services and improve MCH outcomes by recruiting and training PHC workers, renovating facilities, and increasing equipment, drugs, and supply availability [22, 23]. Thus, the demand and supply components of the programme were premised on increasing the utilization of MCH services and ensuring availability of MCH materials, equipment, and essential drugs, thereby reducing the financial burden of healthcare on women community-level awareness was increased through health workers and leadership committees interventions [11, 20].

This paper provides new information based on the demand and supply-side analysis of the SURE-P/MCH services at the primary healthcare (PHC) level in Anambra state, southeast Nigeria, by applying the Three-Delays Model. It shows the factors that affect the provision and utilisation of MCH services, and how the factors can be modulated to improve the provision and access to MCH services. This article should be of relevance to policymakers and practitioners who are interested in creating a sustainable health delivery system.

### Conceptual framework

**Fig. 1:**
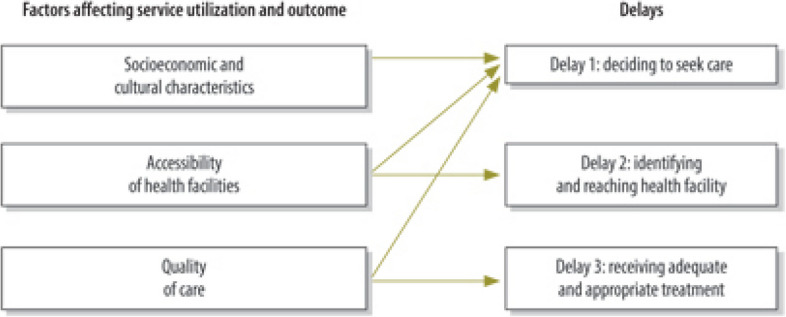
Service utilization and outcome vs. the Three Delays Model. Source: Bulletin of World Health Organization (WHO) [26]

The three-delays model was adapted for the study. This model which was developed by Thaddeus and Maine [24], help to identify community and health services factors (such as those relating to utilization and provision) contributing to maternal deaths and is useful in devising strategies for preventive measures (see Fig. 1). It comprises:

1. *The delay in deciding to seek appropriate medical care* is either due to a previous experience of healthcare, financial implications, low status of women or poor understanding of risk factors in pregnancy and when to seek medical help etc.
2. *The delay in reaching an appropriate source of care is* either due to the cost of transportation, availability of transportation, geography, distance to health centres or poor roads and infrastructure.
3. *The delay in receiving adequate and appropriate healthcare when a facility is reached* is either due to poorly motivated medical staff, inadequately trained medical staff, poor referral system, lack of medical supplies or poorly equipped medical facilities [25–27].

Many researchers have adopted the Three Delays Model. Lawn et al. [28] adopted it in their study on newborns. Some adopted it in their study of understanding the major factors to why newborns die others have used it in studying maternal mortality etc. [25, 29–32]. However, no studies have used this framework to investigate the key factors influencing provision of MCH services towards improving access to maternal and infant care.

## Methods

This paper was developed from a wider REVAMP (DeteRminants of Effectiveness and sustainability of a noVel community heAlth workers programMe in imProving MCH in Nigeria) study [22] which used realist evaluation to examine the effectiveness of the SURE-P programme in Nigeria. It builds on, and complements, analyses of further specific aspects such as security, trust, health worker and village health workers (VHW) motivations, costs and utilisation [15, 33–35] as well as methodological lessons from the research [15, 21, 22]. However, this study did not use the realist evaluation approach. The findings reported in this paper were not predefined at the onset of this study. This paper reports on the emerging themes from the overall study [15, 22, 33–35]. Those themes address the context of this paper (the three-delay model). However, we decided to apply this concept to the providers as well. To understand the factors that cause delays in the provision of MCH services.

### Study setting and sampling

Our study was carried out in Anambra state. According to the National Population Census of 2006 [36], the state has a total population of 4,453,964 and a female population of 2,059,844. Due to the researchers’ long-standing research engagements in the state and in order to have an in-depth understanding of our inquiry into the SURE-P/MCH, this state was purposively chosen. So, from 2012 to 2015, 24 primary healthcare (PHC) facilities were selected to participate in the SURE-P/MCH programme. In October 2012, the SURE-P/MCH programme was implemented in 12 PHCs. Another 12 PHCs were chosen and enrolled in the programme a year later, and ended in November 2015. This study focused on the first 12 facilities because they had been exposed to the intervention for much longer than the other PHCs. Each of these 12 facilities were allocated 6 VHWs. These VHWs (all women) were tasked with identifying pregnant women in the community and encouraging them to use and access MCH services in the community [15].

### Data collection and study population

Data collection was done in two phases. In-depth interviews (IDIs) and focus group discussions (FGDs) were used to investigate the perspectives and experiences of a varied set of stakeholders. Phase one was from March–October 2016 and interviews were conducted by female doctors (EE and CM) and a sociologist (NE) who were trained specifically for the study. In this phase, document reviews were also used to determine the programme (wider study) strategy [22]. The findings of phase one interviews informed the development of eight initial programme theories (IPTs) used in the wider study [14]. In phase two (July–December 2018), interviews were done with other respondents different from those involved in phase one but within the same study population (providers and users of services at SURE-P/MCH intervention facilities). This process was carried out to verify and enhance the wider programme theory based on phase one interviews. Respondents were asked to reflect on the programme interventions from their perspective in each step. Then the investigated theories were consolidated using emerging data from transcripts of qualitative interviews. The use of the three-delay model to analyze the provision of MCH emerged as a distinct theme from the interviews.

In both periods of data collection, we also visited the 12 healthcare facilities to observe the structural security components (perimeter fencing, secure gates, security guards and staff accommodation). All IDIs and FGD respondents were purposively chosen from these facilities. We conducted 35 IDIs with eight policymakers, 10 programme managers, eight facility managers, and eight facility health workers. We also conducted FGDs with eight groups of service users (8–10 respondents per group), eight groups of VHWs (6 respondents per group), and eight groups of Ward Development Committees (WDC) (they are community representatives in charge of overseeing the management of the facilities) participated in focus groups (6–8 respondents per group). Nurses, midwives, and community health extension workers (CHEWs) are examples of health workers (all females) [15]. All interviews lasted between 40 and 60 min. For this research, interview question guidelines for several groups of respondents were created, and are available on request.

### Data analysis

Interviews were transcribed verbatim by the researchers and grammar checked, coded, and analysed (both manually and using NVivo 11 software) into themes. The coding was inductive. The lead researchers developed a codebook, Using NVivo 11 and the codebook, transcripts were coded individually by two researchers, who then came together to agree on any disparities. The codes were generated to focus on supply and demand side analyses, respectively. The main themes were identified from the transcripts, then colour-coded and analysed. These themes emerged from the overall study [22]. The findings of this study are not pre-defined but emerged as themes. At several phases of the process (tool piloting and post-piloting review, collection, transcription, translation, anonymization, digitization or entry into software, coding, and analysis), the quality of data collection was assured. Multiple quality assurance techniques were implemented, including proper training (e.g., of transcribers of core concepts/terms used) and multiple researchers working on the same data (e.g., coding by at least two researchers). During analysis, we adapted the third delay of the three delay model to focus the delays from the Supply side.

## Results

The Three Delay model was designed to understand the delays that affect health utilization (demand).

## Key factors that delay utilization

### Demand – three delays model

#### First delay

##### Previous experience: the delay in deciding to seek appropriate medical care

Some service users found it difficult to decide to visit the health facility. This is because several of the facilities were in poor condition before the SURE-P. The facilities had not been refurbished; there was no security. This created a sense of uncertainty, anxiety, and insecurity among the service users, resulting in the issues raised by the respondents. For instance, pregnant women and their families have been turned away from unsecured facility doors because night duty personnel were unable to identify potential service users and did not feel comfortable opening the doors. Also, there was no staff-patient relationship. There was a facility where the officer in charge (OIC) did not have a good relationship with the service users. These delayed their decision to seek care at the facility. A quote from one of the respondents expressed this:

*“That is the most important thing because when a pregnant woman is in labour and you are scolding her or telling her irritating things, it may cause the woman and her husband to start facing another thing from what they know not after delivery. So I want them to try more than the way they have been trying before… I am saying from what I witnessed here. I don’t know what has been happening here; I say things I witnessed.” (Family member of service user, FGD participant 7)*

Another respondent speaking on a previous experience she had that delayed her decision to seek appropriate medical care said that,

*“…my second issue was here although my husband resisted at first saying that they don’t take care of people well. It was my mother-in-law that made him change his mind by telling him that the maternity he once knew was no longer the same. When I delivered here I discovered that they were good in all these; that is, they were not lacking in any way so I decided that I will have my third child here as well.” (Service user, FGD participant 7)*

#### The second delay

##### Cost of transportation and geography: the delay in reaching an appropriate source of care

The second delay refers to how the road is (are the roads bad?), and how long it takes to get to the institution that offers emergency obstetric care. The cost of transportation to the facility is another key factor that causes a delay in the utilization of MCH services. The cost of transportation concerns prevents these women from getting to an appropriate source of care on time. The route from the home of the service users to the facilities was bad *“not just bad but horrible”*. Sometimes, if unable to get any means of transport*, “one would have to trek to the facility” (Service user, FGD participant 1)* or stay home until they have transport fare. So, this second delay is then initiated because of the difficulty in getting transport, the cost of transportation and the long travel distance to the health facility experienced by the service users. A respondent said this:

*When I don’t have transport fare I stay put for that week… For me, I weigh these sometimes and stay put when I don’t have the money. So, sometimes I delay my visits to enable me to gather enough money for up to 2 to 3 weeks before I eventually come for another visit. (Service user, FGD participant 2)*

#### Third delay

##### Lack of equipment and poor referral system: the delay in receiving adequate and appropriate healthcare when a facility is reached

The lack of equipment in most of the facilities discouraged some of the service users from visiting the facilities, despite pressure from family members, they still refused to use these facilities, stating that they were poorly equipped. *“I do not like the idea that this facility is ill-equipped.” (Family member of service user, FGD participant 7).*

## Supply – three delay model

### Key factors that delay the provision of MCH services

#### The delay in deciding to provide appropriate medical care

In our study, we found that facility security was a factor that delayed the provision of MCH services. We explored the presence or absence of security components during the programme, and community efforts to maintain facility security after the programme. The absence of security (fence, security guard and adequate lighting) within the facility made health workers feel unsafe within the facility, especially at night, with a resultant reduction in 24-h access and utilization of facility services by service users. Before the SURE-P MCH programme, some facilities were not fenced and did not have gates and security men to safeguard health workers and patients. This created a feeling of fear and insecurity among the health workers and service users, which resulted in challenges highlighted by the participants. Quotes from a health worker and Village Health Worker (VHW):

*“When I was there, there was an incident, It wasn't a case of a pregnant woman anyway, but the client came by 2:00 am and I was so scared of opening the gate for them because there was just a lady and up to five guys, so I had to tell them to go down the road and call the security men on the street to accompany them before I can open the gate, so security is really necessary.” (Health worker, IDI participant 1)*

“*The nurses were scared of lack of security man then, and the women started reporting to us that some women in labour had gone there at night, and they stayed and stayed without seeing anybody, so they left to another hospital. So, it posed a very big challenge then.” (VHW, IDI participant 1)*

#### Transportation and geography: the delay in reaching an appropriate source of care,

The second delay that affects the daily running of the facility is the poor citing of the PHC (remote location and poor roads). Finding a vehicle that would take the health worker (from their home to the facility and back) was difficult. Especially, leaving the facility, sometimes the health worker had to trek to the “main road” where they could then find a vehicle (*okada – a motorcycle taxi* or *Keke – a tricycle)* to either go for errands or to go to the LGA*.*

*“…you know this place is a bit inside… So is only Okada that comes here… I think the road is also bad.” (Health worker, IDI participant 2)*

#### The delay in providing adequate and appropriate MCH care

Using this third model for providers, we also explored the delays that arise in the provision of adequate and appropriate healthcare. We found that these facilities lacked means of transportation to convey service users with complications. Also, we found that there was a lack of trained staff to manage equipment, no ambulance (for referrals), poor electricity supply, poor water supply and inadequate accommodation.

*“…at times going to the hospital during referral is a problem. There was a day a woman had a problem, Uterine prolapse, no vehicle to carry the woman to the hospital, it was the vigilante that I saw that carried the woman to the hospital.” (Health worker, IDI participant 3)*

*“Some equipment that is needed has been provided and they are there. They are in this facility, although in some cases, there may not be anyone to manage [operate] them. Like in the laboratory department, I have equipment in the laboratory, but there is no technician to manage it, so there are some gaps there.” (Facility manager, IDI participant 1)*

## Discussion

**Fig. 2:**
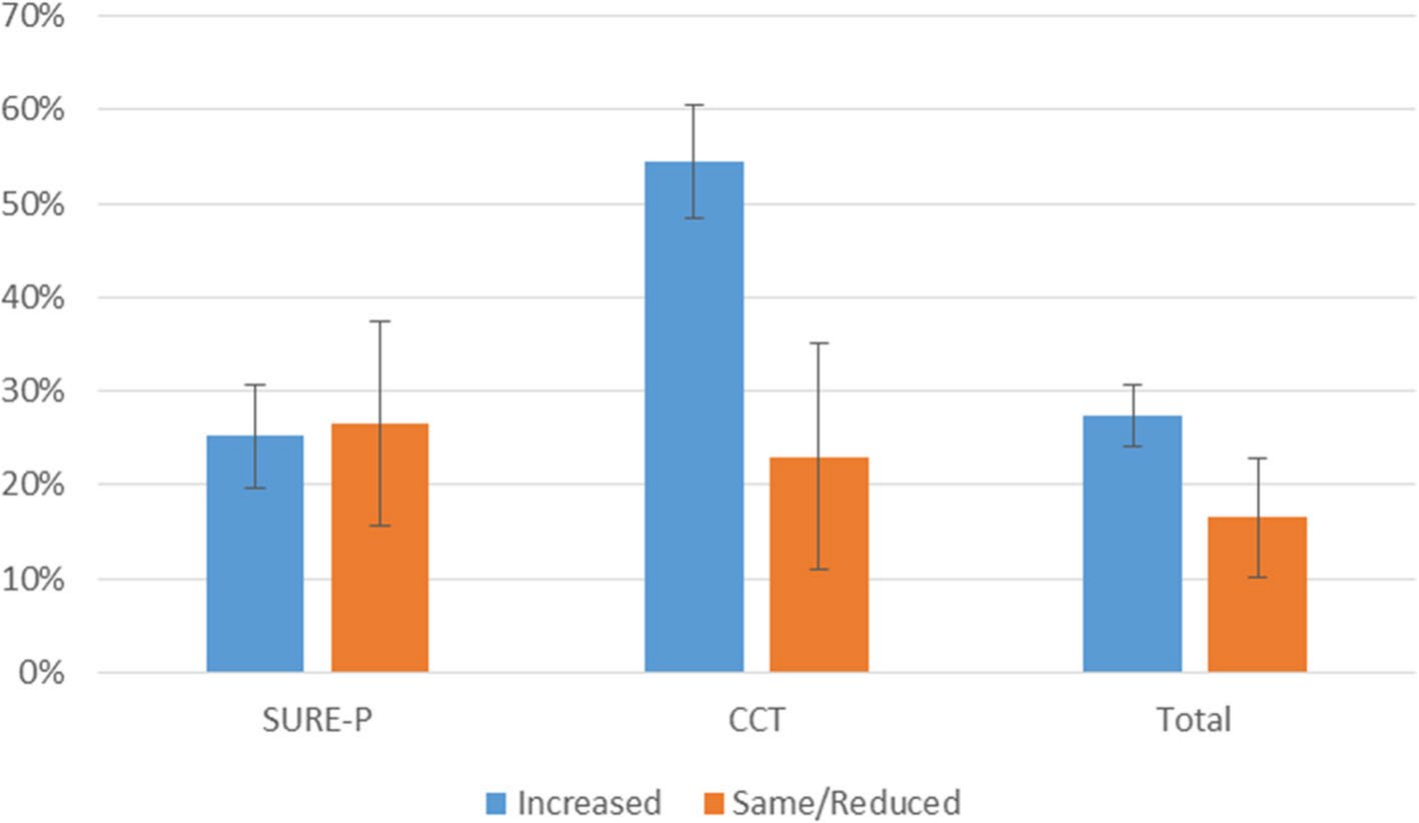
Change in trust once SURE-P was introduced (% of women) *(source: Role of trust in sustaining provision and uptake of maternal and child healthcare: Evidence from a national programme in Nigeria)* [33]

The SURE-P programme's main goal was to increase the use of maternal services in public facilities, particularly among the poor. The population in the target regions were mostly rural and according to a household survey conducted throughout the study, antenatal care is almost universal, at 99%. (unpublished data from the REVAMP project). Delivery in public health facilities was around 57%, with the remainder delivered by private providers or at home [33]. Our findings revealed an increasing trend in MCH service utilisation in the CCT cluster [33] from 2012 to 2015. There was a consistent upward trend in all six MCH indicators: antenatal attendance, antenatal first visit, antenatal fourth visit, skilled birth attendant delivery, pregnant women receiving their second dose of tetanus toxoid, and the number of children fully immunised at one year of age. The introduction of SURE-P/MCH improved women's trust in the health system, with 25% of those living in SURE-P/MCH regions and 55% of those living in CCT areas reporting that their trust in the health system had grown as a consequence of the resources offered by the SURE-P/MCH programme (see Fig. 2) [33].

The three-delay model serves as a framework for this discussion. The factors that fall under the categories listed under this model are considered key and seen as causing delays that could lead to maternal or infant mortality. Several studies have discussed barriers to utilisation, factors that affect utilisation, etc. While the current study touches on these subject matters, the focus is, however, on the key factors that are impactful enough to cause delays in both MCH service utilisation and provision.

The first delay occurs at the household and societal or community levels, and it reflects the delay in deciding to seek medical attention for pregnancy issues [24, 32]. Aside from the quality of services provided by a health facility, patients' experiences and perceptions of quality care affect health facility utilisation. A woman's opinion of quality care may influence the behaviour of another individual seeking health care [37, 38]. For example, if a woman had a negative experience with care during a normal delivery, other women whom she may have recounted her experience to may have delayed seeking care, thus increasing the likelihood of birth complications and maternal death [37–39]. In our study, this experience could be the attitude of the staff, the way they treat people, or the nature in which they offer treatment. We found that there were cases where the staff did not treat their patients with care, which led to these patients deciding not to seek medical care in such a facility again. Thus, if this facility is near any of these patients who decide not to utilise their services in an emergency, such a delay could bring about complications that might ultimately lead to death. Medical facilities must prioritize patient care and ensure that all providers are trained to provide compassionate and attentive treatment. Neglecting patient needs may have severe consequences, especially in emergencies where timely medical intervention is vital. Therefore, healthcare providers should address any shortcomings in staff attitude and behaviour to prevent potential life-threatening outcomes. In a study by Mgawadere [40] In Malawi, it was found that out of 151 maternal deaths, only 32 (16.6%) occurred at home, and these deaths were caused, by perceived poor quality of care at the health facility, an uneventful previous delivery, and a lack of awareness of obstetric complications. These were found to be the main reasons given by the women’s families for not seeking care. Lack of trust in the health workers leads to delays in demand for appropriate MCH services. Thus facilities without any forms of security lead to both demand and supply constraints. Similar findings have been reported elsewhere [15, 41, 42].

The three-delay model has never been used to analyse MCH provision. So, for this first delay, the providers also had delays in deciding to provide services. When a service user visits the facility at night and the facility does not have a fence, a security guard, or even adequate lighting (outdoor lights/lamps), the providers will be sceptical about whether to let them in or turn them away. These facilities are staffed by women, and opening the door at night is extremely dangerous, especially if the service user is accompanied by men. The safety and security of both the service users and the providers are of utmost importance. Without proper measures in place, such as a fence, a security guard, or adequate lighting, the providers may face increased risks and concerns about potential harm or violence. Therefore, it becomes crucial to prioritize the implementation of safety measures to ensure a safe environment for everyone involved [15]. This delayed their decision to provide MCH services. The delayed decision to provide MCH services highlights the significance of addressing safety concerns. By taking the necessary steps to establish a secure environment, the providers can confidently offer their services without compromising the well-being of both service users and themselves. Additionally, prioritizing safety measures not only protects against potential harm or violence but also fosters a sense of trust and reassurance among all parties involved, ultimately enhancing the overall quality of care provided.

In the second delay, the delay in demand to reach an appropriate source care was found to be caused by the cost of transportation, the geography of the roads that lead to the facility and security concerns. For the service users, while the cost of transportation causes delays, the poor roads leading to the facilities pose a bigger delay for them. They find it difficult to get a transport that will convey them to facilities, and when they are not able to find one, they stay home. This delays their reaching an appropriate care, which could lead to a home birth. In addition, the lack of reliable transportation options also impacts the availability of emergency medical services for service users. In cases where immediate medical attention is required, the poor roads make it challenging for ambulances or other emergency vehicles to reach the facilities promptly. This further exacerbates the potential risks and complications associated with home births. For the providers, they also have the same issue. As many of the facilities do not have staff quarters, they have to transport themselves from their residential areas to the facilities. Even if they find a vehicle that will take them to the facility, leaving the facility after the afternoon shift can become daunting (the same goes for the users). If a service user reaches the facility and the provider is not available (because she has yet to find a vehicle to take her there), it can lead to frustration and a delay in receiving the necessary care or treatment. The poor roads (especially during the rainy season) can further exacerbate the transportation challenges faced by both service users and providers. The deteriorating road conditions can make it even more difficult to find a vehicle willing to navigate through the muddy and pothole-ridden roads, adding to the already limited transportation options available. This lack of transportation options and poor roads not only delays the accessibility of healthcare services for both users and providers but also highlights the need for improved infrastructure and support systems to ensure seamless access to healthcare facilities.

In the third delay, which is barriers to the receipt of timely and appropriate obstetric care at the facility level, there are six factors involved. These factors are grouped as follows: drugs and equipment factors; policy and guidelines factors; human resources factors; facility infrastructure; patient-related and referral-related factors [29]. In our findings, after one decides to seek medical care and finally reaches an appropriate source of care at the facility, they face another delay in receiving adequate and appropriate care, which is worrisome. The users reported that the facilities were ill-equipped and lacked drugs. In addition, the provider reported that when there is a complication, finding the vehicle that will transport the client is very difficult. Dalinjong [43] found that only 7% of the facilities studied had emergency transport for referrals, and basic drugs, supplies, equipment, and infrastructure were inadequate. The poor healthcare infrastructure further exacerbates delays in receiving appropriate care. The users found that not only are the facilities ill-equipped, but they also face a shortage of essential drugs. Moreover, the providers mentioned that in cases of complications, arranging transportation for the client becomes a major challenge. These issues highlight the need for significant improvements in the healthcare system to ensure timely and effective care for patients.

The study shows that more emphasis is needed to address the availability and quality of services within existing service delivery platforms so that the local service delivery system adequately meets the needs of the population.

### Study strengths and limitations

The strengths of this study include its comprehensive analysis of the healthcare infrastructure and its impact on patient care. The inclusion of both user and provider perspectives adds depth to the findings. However, it is important to acknowledge that this study has some limitations. For instance, the findings may not be generalizable to other healthcare systems or regions due to the specific context in which the research was conducted. Further research with larger and more diverse samples or methods would be beneficial to validate these findings.

#### Policy implications

The three-delay model should be used as a planning tool by policymakers to ensure that an optimal balance of supply and demand is delivered within an existing health system and that links between community and facility levels are sustained. By applying this framework, policymakers and healthcare providers can identify specific areas for improvement and develop targeted interventions to address the underlying issues. Additionally, this approach can help build trust between women and health workers, ultimately reducing delays in seeking and receiving essential MCH services.

## Conclusion

This model examines delays in decision-making, accessing care, and receiving (providing) appropriate treatment. Our findings show that there were problems with both the demand and supply aspects of the programme, and both were interlinked. For service users, their delays were connected to the constraints on the supply side. There are some issues related to optimal supply and demand, and addressing these issues will require a comprehensive approach that considers both the demand and supply aspects of service delivery. This could involve improving infrastructure and resources for healthcare facilities as well as implementing strategies to increase awareness and utilisation of MCH services among the target population. Additionally, establishing effective referral systems and ensuring timely access to emergency transport can help address the constraints on the supply side and reduce delays for service users.

## Data Availability

All dataset used or analyzed during the current study, are available from corresponding author on reasonable request. The interview and focus group discussion guides were developed as part of the study and also, have previously been published in Etiaba [15] and Ebenso [14].
